# Ammonia Treatment of Rheumatism

**Published:** 1877-10-20

**Authors:** Meriwethe Lewis

**Affiliations:** Lenoir’s, Tenn.


### AMMONIA TREATMENT OF RHEUMATISM.

By MERIWETHE LEWIS, A.M., M.D., Lenoir's, Tenn.

Some months since, quite a stir was made in the journals on the above mentioned plan of treating rheumatism, and it was generally considered entirely new. Every one will no doubt remember that it was claimed that a few six drop doses of aqua ammonia would relieve a case of rheumatism in the course of six or twelve hours.

In the winter of 1873, (February) I was treating a case of sub-acute articular rheumatism in the person of an old lady. I sent her a prescription of iodide of potassium and tincture of aconite, directing twenty drops to be taken every three hours. Volatile liniment was also ordered for rubbing the joints. By some strange mistake, the messenger, her son, reversed the method of using the prescription, so as to give her twenty drops of the liniment, while rubbing was vigorously carried on with the iodide and aconite.

Seeing her in a few days, I found her entirely well; and although enthusiastic in praises of the "hartshorn" she had been taking, she nevertheless pronounced it to be the "most abominable stuff she had ever tasted." On enquiry I found that her improvement began soon after taking the first dose. Only three doses were taken that day, and one the next.

I was too wise to explain that there had been any mistake, but *per contra*, profiting by it, I have since used the aqua ammonia in a few suitable cases of rheumatism, and so far, with most flattering success.

In conclusion, I would suggest that the remedy is worthy of a trial; and I do not doubt but that others will meet with equal success in its employment.

